# Medical and Physical Intelligence

**Published:** 1799-11

**Authors:** 


### MEDICAL AND PHYSICAL

### *INTELLIGENCE,*

### ( Original and Selected. )

*Observations on Soda, the Alkaline Basis of Animal Gall and of Sea-Salt, and the Effects it produces in the Concoction of Food, and in preserving Provisions; in a Letter from Dr.* MITCHILL to JAMES WOODHOUSE, *M. D. Professor of Chemistry in the University of Pennsylvania, dated New-York, Dec.* 23, 1798.

FROM the facts stated in his former essays, Dr. MITCHILL thinks the antiseptic powers of alkaline earths and salts are incontestibly established. These substances seem to have been produced, in the œconomy of nature, to make a stand against that predominant acidity, which, without their interference, would threaten the animated world with ruin.

But alkaline substances are not only strewed over the face of the earth to exercise their neutralizing operation there; *soda* exists in the waters of the ocean, combined with muriatic acid, and in the gall of animals, united to an inflammable resin. And both sea-water and bile excite, though in different degrees, a bitter taste.

Many animals that have plenty of gall are greedy of sea-salt; even wild creatures in the American woods can be tempted by it to approach a man who carries salt in his hand. The instinctive appetite for this substance is a very remarkable thing, and is worthy of particular consideration. Sea-salt has been denominated *muriat of soda*; and for the sake of uniformity, and to make the subject a little clearer, Dr. MITCHILL, for want of a better name, and without contending for the propriety of it, calls bile, or gall, the *bitter of soda*. It cannot be improper to make an approximation in terms, where there exists a strong analogy in nature.

Dr. M. remembers, ever since his childhood, the custom of American bandmen to save the gall of the beeves they slaughtered for family use in autumn. A piece of the liver was cut out with the whole gall-bladder, and generally hung up on a neighbouring tree. Perhaps it was carried into the kitchen and stuck up there, or, perhaps, remained in the tree until the next summer. In either case, whether exposed to culinary warmth or wintry cold, the contents of the cyst underwent no sensible change, saving a thickening by the drying away of its moisture. There was never any putrefaction or sign of taint. The use for which they kept it was for plaisters to put on the feet of their children and labourers when wounded by nails and thorns; and Dr. M. questions whether pharmacy can furnish a better one.

In respect, then, to its uncorruptibleness, the bitter of soda has a near resemblance to the muriat of soda; that is to say, it will remain an indefinite definite time, in expofure to common atmofpheric agents, without undergoing decompofition.

Surely, then, the *acrimony*, the *putridity*, the *cauflicity*, and the *numberlefs mifchievous qualities* afcribed to bile by modern writers, are not real conditions of that liquid, but notions of their own heads. Far different from fuch falfe and fabricated ftatements is the action of that *natural foap*. Mild, wholefome, and incorruptible, the bitter of foda ftands ready to check the growth of acidity, and limit the progrefs of the alimentary mafs in its tendency to corruption.

If diforders of the alimentary canal are fometimes accompanied with a great flow of the bile, the bitter of foda flows to wafh away or neutralize the offending caufe in the ftomach or duodenum. As a mote in the eye ftimulates the lacrymal gland to fecern tears, and prevent its injurious effects, fo an acid ftimulus in the primæ viæ excites the liver to prepare and fend forth a fufficient quantity of the bitter of foda to overcome it. Such is its kind and pleafant operation. Never has Dr. M. found any thing like an experimental proof of its injurious qualities. Yet the mifchievous effects of fubftances fwallowed, and corrupting in the inteftines, have generally been afcribed to the bile, which is fpoken of with a vehemence and repetition of abufe that hardly any thing could exceed. As well might the naufea and giddinefs from a quid of tobacco be afcribed not to the herb, but to the fpittle which it ftimulated the falivary glands to pour into the mouth. With as much propriety might blifters of the *fkin* be afcribed to the ferous fluid effufed by the exhalent arteries, and not to the epifpaftic of cantharides.

Experiments have abundantly fhewn, that muriatic acid, poured upon bile, combines with the foda, and conftitutes common falt. In thefe experiments, the bitter of the bile is precipitated or fet loofe. It does not yet appear to be quite fettled wherein *bitters* exactly differ from *aftringents*. With refpect to their compofition, Dr M. fufpects the difference is rather in *degree* than in *kind*. If fo, as *aftringency* confifts in the combination of the gallic acid and the earth of alum, there can be conceived nothing injurious to health in that portion of the fecretion of the liver.

If muriat of foda can be thus produced artificially, and *out of the animal body*, there can be no doubt of the poffibility of its formation by a natural procefs *within* the body, where the fpirit of falt is taken as an article of food or of medicine. Glauber feems firft to have practifed the extraction of this fingular and moderate acid from its alkaline bafis in fea-falt, (2 Boerhaave Chem. Proc. cxliii.) and to have given an account of its excellence in diet and domeftic œconomy. Boerhaave defcribes it as "remarkably agreeable to the ftomach, promoting appetite, attenuating mucus, affifting digeftion, preventing putrefaction," &c. and concludes the character of this liquid procured from fea falt by affirming, that "it is above all praife." (Ibid.) And Hoffman (Obs. Phys. Chem. Lib. 2, Obs. xvii.) fpeaks of the ufing of fpirit of falt with flefh broths as a common practice. It is rather a pity, that the oxygenated forms of this acid and their combinations have chiefly attracted the notice of the more modern chemifts.

Yet, though the fpirit of falt is fo fafe and valuable a preparation, the untutored appetite of man and other animals has led them to prefer the muriatic acid in conjunction with foda, that is, fea-falt or common falt, to the fpirit of falt itfelf. There muft be a reafon for this; and this reafon, reaſon, which is founded deep in the nature of things, is worthy to be ſearched for.

Thus the conſtitution of many creatures is ſupplied not only with the *bitter of ſoda* furniſhed by the liver, but with the *muriat of ſoda* taken in with the food. Then the queſtion to be decided is, What are the chemical effects of the muriat of ſoda taken into the ſtomach with the aliment? Now, in creatures who eat ſubſtances which contain ſepton, or the baſis of the acid of putrefaction, there may happen caſes of ſurcharge, retention, or indigeſtion; and, at ſuch times, may be formed within the ſtomach or inteſtines a portion of that offspring of corruption, the ſeptic acid, after the ſame manner as in other putrefactive proceſſes. Beſides the agreeable ſtimulation afforded by the muriat of ſoda to the palate and ſtomach, its alkali lies ready to ſeize and neutralize any little parcel of ſeptic acid that may happen to be produced. After this manner, that injurious acid is taken out of circulation, and the mild and ſalubrious ſpirit of ſalt is extricated in its ſtead. And, in a ſimilar way, the ſoda of ſea-ſalt has an operation in the ſtomach ſimilar to the ſoda of the gall in the inteſtines. In the former caſe, the muriatic acid is ſet looſe—in the latter, the bitter principle. In both the ſoda will quit its preſent connections, to combine with a ſtronger acid. In ſuch caſes cubic nitre is the new product. Where no acid is generated, there will be no decompoſition of either. The reaſon, then, is apparent, wherefore it is better to take the muriatic acid *with ſoda* than without. There may be a farther uſe, that from this ſource the bile itſelf may derive its alkali.

Yet the muriat of ſoda is not only mingled with food when eaten, but many ſubſtances intended for nutriment are ſprinkled, pickled, or impregnated with it in the larder and kitchen, before they are ſerved up at table. Sea-ſalt is preferred for culinary purpoſes, not becauſe it is the *ſtrongeſt*, but the moſt *palatable* and *agreeable* of antiſeptics. This may be beſt underſtood by ſhewing what ſoda can do alone.

Dr. MITCHILL thinks, the operation of this alkali to overcome the harmful products of putrefaction, is amply ſupported by the ancient mode of embalming, as practiſed in Egypt. For HERODOTUS, in deſcribing the proceſs, particularly mentions, among other things, that the bodies WERE SALTED and hid in NITRE for ſeventy days, (B. ii. cap. 85, 86.) "the uſe of which was to "dry up all their ſuperfluous and noxious moiſture." The Greek hiſtorian uſes both the word νιτρον and the atticiſm λιτρον, to expreſs the ſubſtance uſed for ſalting the bodies. Now, it is a ſettled point, that theſe words, though tranſlated "nitre," mean not the "ſalt-petre" of the moderns, but ſoda, or the baſis of ſea-ſalt. This chemical correction, ſtrengthened by the authority of WARBURTON, (3 Divine. Leg. b. 4. § 3.) contending that the body of ISRAEL (Geneſis, c. l. 2, 3.) was kept by the embalmers the cuſtomary time in nitre, (carbonat of ſoda) evinces how long ago it was known that the mineral alkali abſorbed the ſeptic fluids produced during the corruption of human bodies.

[To be concluded in our next Number.]

### *Experiments with the Eudiometer, made at Martinique, by* Dr. George Davidson.

[Communicated to Dr. Mitchill, in a Letter, dated Fort Royal, April 7th 1798.]

THERE is a large proportion of oxygen contained in the atmofpheric air within the tropics. Having by accident broken the eudiometer which I had borrowed from Mr. Baker, I wrote to my friend George Wilson, of Bedford Street, to fend one to your addrefs, and one out here. I have fince repeated the experiment, both in the town of Fort-Royal, and the neighbouring heights. With equal meafures of nitrous air, obtained from brafs wire and atmofpheric air, there was an abforption of 67.100; but with two meafures of atmofpheric air, there was fomewhat lefs, from 52.100, to 58.100, that is to fay, from three meafures, or three hundred parts, there was left two and 42.100, to 48.100. I have alfo tried a mixture of fulphur and iron-filings, in a cylindrical glafs tube, and found above four-tenths of the air diminifhed. As thofe experiments were made in the prefence of a number of medical gentlemen, who have certified the fact, as they have obferved it themfelves, I can therefore with confidence fpeak to the refult of our experiments.

We fhall now be more eafily able to account for the fpeedy rufting of metals, without having recourfe to the action of fuppofed muriatic foda diffolved in the air. The rancidity of oils, and the fpeedy fermentation of vinous liquors, may, perhaps, be alfo owing to the greater heat of the climate; but metals would be lefs apt to contract ruft from the greater heat, if the air contained only the proportion of oxygen, which it contains in Europe. Confumptions here are alfo more quickly fatal than they are in Europe. On fome of the Weft India iflands, fuch as Barbadoes and Antigua, where lefs rain falls, and where the heat of the air is kept at a fteady temperature by their fmallnefs, and the trade-winds blowing over the whole ifland, without being diverted by high or woody land, confumptions are not lefs fatal than in the larger and moifter iflands. This is a fact, of which all writers upon difeafes of the Weft Indies take notice; and which we would principally endeavour to account for, from the air being more highly charged with oxygen, than it is in Europe. If people labouring under pulmonary confumption, are fome times benefited by fea voyages within the tropics, we are to look for the temporary relief afforded in the fea-ficknefs; the irritable ftate of the ftomach, by fympathy preventing the the lungs acting in decompounding and abforbing oxygen; and in the determination to the furface carrying off the redundancy by the fkin. Their coftive habit, whilft at fea, proves that there is more than ufual determination to the furface—hence relief to the lungs.

To

*To this Letter are ſubjoined the two following Certificates; the reſpectability of the witneſſes, places the beforementioned facts beyond every doubt.—The firſt is from* Dr. Chisholm.

[Within two miles of Fort Royal, upon an elevated ſituation.]

Dear Sir,

IT is with pleaſure I comply with your requeſt, reſpecting the proportion of oxygen in the atmoſphere at this place, as aſcertained a few days ago, by means of your eudiometer, and by a compoſition of ſteel filings and ſulphur. By the firſt, the quantity I think was about 56.100: for although 57 was given by one trial, yet the medium of all thoſe made was no more than 56. A ſimilar reſult was given by the common glaſs tube—a proof, I ſhould imagine, of the accuracy of the experiment made with the eudiometer. The reſult of the trials with the filings of ſteel and ſulphur, I think, was about 40.100 parts. It appears to me to be a ſingular circumſtance, that, although the ground on which the Ordnance Hoſpital ſtands is a perfect moraſs, partially drained, yet a reſult almoſt exactly ſimiliar to that given by the experiments made with the eudiometer at my houſe, ſhould take place, with the ſame inſtrument, and under circumſtances in no way different. The proportion at the Ordnance Hoſpital, I think, has been 58.100; and at your houſe in the Grand Rue, a ſituation leſs ſwampy, and nearer the ſea, it has been 67.100.

The ſituation of this place is high, dry, and enjoys as pure an air as it is poſſible to have in this country. An explanation of ſo ſingular a reſult, in ſituations ſo different, is perhaps more to be wiſhed for than expected.

I am, &c.

C. Chisholm, M. D.

*Inſpector of Ordnance Hoſpitals.*

Dr. Davidson, *Fort Royal.*

*The ſecond is from Capt.* Harvey *and Surgeon* Lindesay.

WE whoſe names are hereunto ſubſcribed, do certify, that we have ſeen the experiments mentioned in the former part of the letter, upon the compoſition of the atmoſpheric air; which were made both with Fontana's eudiometer, and a cylindrical tube with nitrous air, obtained from the diſſolution of braſs in the nitrous acid; and that we found 57.100 of vital air abſorbed in the former, and above half by the latter.

W. M. Harvey, *Captain,* } *Firſt Weſt India Regt.*

John Lindesay, *Surgeon,* }

*Miſcellaneous*

### *Mifcellaneous Extracts.*

Profeffor GÖTTLING makes the following remarks *on the Origin of Carbon in confequence of treating Phofphorus with the Carbonat of Soda :*

HE firft obferves that he has formerly, in his Chemical and Pharmaceutical Almanack, for 1796, mentioned the decompofition of phofphorus by the carbonat of lime, and the carbonat of foda, whence are produced carbon and the phofphat of lime, or mineral alkali. It is certainly true, that carbon will thus be obtained, and it is not even neceffary to inclofe phofphorus with calcareous earth, or foffil alkali, in glafs tubes ; there is nothing farther required than to place a piece of phofphorus in a fmall glafs veffel, to add a fmall portion of mineral alkali, dried in the air to a powder, and heat it gradually on a charcoal fire, till it become red hot: it is, however, neceffary to increafe the heat of the fire, by blowing it with a pair of bellows. The phofphorus will thus be kindled, and the greateft part of it confumed, without producing any farther effect on the mineral alkali. The refiduum is of an uniform black colour; and if its falt be diffolved in water, there remains an infoluble carbon, of a deep black colour. The carbonat of potafs may be employed for the fame purpofe, and with fimilar effect. GÖTTLING's *Almanach für Scheidekünftler und Apotheker,* 1798, p. 1—3.

---

*On the precautions neceffary in melting the heavy fpar,* the fame author obferves, that in melting this fubftance with the carbonated alkali, to feparate the barytes, it is neceffary to proceed very flowly ; as the mixture, in a glowing ftate, fhoots out with violence, and renders the attention of the operator dangerous. A tough cruft is formed on the furface of the melting mafs ; and if at the bottom of the glowing mixture any humidity fhould remain, which could not efcape during too quick a procefs, this circumftance may occafion the fudden expulfion of the uppermoft cruft in all directions, as the fluid affumes the elaftic form of vapour.—*Ibid.* p. 13.

---

When Prof. GÖTTLING, in his laft courfe of chemical lectures, was fhewing by experiment, the ufual mode of preparing the *Bononian phofphorus,* by expofing to a glowing heat gum tragacanth, and barytes reduced to powder and carefully feparated from its iron, he obferved that all the fmall cakes committed to the fire were melted into one mafs, of the nature of enamel. It is well known that the ufual phofphoric Bononian ftones are more porous, and, if properly prepared, emit a red light in the dark, after having abforbed the rays of light during the day. Thofe, however, which the Profeffor obtained by the aforementioned procefs, afforded a white light, and acquired their luminous property in a very uncommon degree, by means of the electric fpark.—*Ibid.* p. 14.

---

As the *yellow colour of the muriatic acid* is unqueftionably owing to the *particles of iron* which it contains, the contrary effect taking place in many accurate chemical experiments muft be afcribed to that ingredient. To effect, therefore, a feparation of the iron, Prof. GÖTTLING adds to every every pound of the common muriatic acid, free from the vitriolic, half a drachm of a dry pruffiat of kali. The fluid foon affumes a perfectly blue colour, and afterwards gradually acquires the colour and tranfparency of pure water; depofiting at the bottom of the veffel a confiderable portion of Pruffian blue. After this, the clear fluid is poured into a tubulated retort, and diftilled over till about one ounce remains. Thus the rectified acid becomes perfectly white, without containing the leaft particle of iron.—*Ibid.* p. 6.

---

The pharmaceutical operator is fufficiently acquainted with the difficulty of combining *lard*, or *fat*, *with quickfilver*, by trituration. This procefs may be much expedited, by adding a very fmall proportion of the flowers of fulphur to the mercury; a difcovery lately communicated to GÖTTLING, by M. BERNSTEIN, an eminent furgeon in Germany. A mixture confifting of two ounces of hog's-lard and fix ounces of quickfilver, requires only fix grains of the flowers of fulphur, and the combination will take place in a few minutes. As fo very fmall a proportion of fulphur is not likely to affect the efficacy of the ointment, this practical hint deferves the attention of the pharmacopolift.—*Ibid.* p. 15.

---

As the ammonia checks the *luminous property of phofphorus in azotic gas*, it is obvious that phofphorus will not appear luminous in gas obtained by depriving atmofpheric air of its oxygen, by the hepatized ammonia; becaufe, in this procefs likewife, a fmall portion of ammonia remains behind in the azotic gas. But if, for this purpofe, the fixed hepar fulphuris be employed, the phofphorus will uniformly appear luminous. Prof. GÖTTLING obferves, that he has kept azotic gas prepared from atmofpheric air, for a whole year, over a folution of the liver of fulphur, which, in eudiometrical experiments, does not manifeft the leaft trace of oxygen, and which, neverthelefs, retains undiminifhed its power of producing the luminous appearance of phofphorus.—*Ibid.* p. 16.

---

The late Prof. GREN, of Halle, confiders in his "Elements of Phyfics," third edition, 1797, *the combuftion of phofphorus* as a gradual and flow procefs, which, in atmofpheric air, though operating flowly, affords *the fafeft and moft regular eudiometer*. He defcribes the neceffary apparatus and regular procefs in the following manner: "An accurate cylindrical glafs tube, with one end clofed, muft be procured, and divided by a fcale into a fufficient number of fmall parts, at equal diftances. This tube is filled with diftilled or rain water; the air to be examined fhould be introduced in a tub, under water, and the quantity carefully marked, together with the ftate of the barometer and thermometer. Several needles are to be paffed through a cork-ftopper of a fmaller diameter than the tube; on the projecting points of the needles is fixed a piece of pure and tranfparent phofphorus, while a linen thread is faftened to the lower end of the cork. This cork is placed under the mouth of the glafs cylinder, in the water of which it will afcend, and the phofphorus on it will come in contact with the air contained in the cylinder. The apparatus is placed in a proper veffel with water, in which it is left ftanding. The phofphorus gradually melts, while it is luminous, and the cork may, from time to time, by means of the thread, be drawn under the water, to waſh away the acid adhering to the phoſphorus, and thus render it more active. When, at length, all the oxygen gas is conſumed, and when the remaining phoſphorus is no longer luminous, the cork ſhould be withdrawn, and the exact reſidue of the azotic gas, as well as the conſumed oxygen gas, ought to be marked with the correſponding preſſure of the barometer, and the degree of heat obſerved by the thermometer."

It muſt be admitted, ſays GÖTTLING, that this apparatus is very ingeniouſly contrived, but it would ſtill be unſafe, if the remaining air ſhould not be pure azote, but azotic gas filled with phoſphoric vapours, and if that air ſhould occupy a greater ſpace than azotic gas alone; it is, at leaſt, proved by the ſmell of this column of air, and the vapours ariſing after the admiſſion of atmoſpheric air without the preſence of phoſphorus, that this body of air cannot be pure azote.—*Ibid.* p. 24—27.

---

M. SCHWARZ, a German apothecary, has lately obſerved, that, by diſſolving ten grains of the nitrat of mercury in one ounce of diſtilled water, and adding two drachms of the mucilage of gum arabic, the nitrat is completely decompoſed, while a copious grey precipitate is formed. M. JUCH, who was preſent during the experiment, is of opinion, that this decompoſition muſt be aſcribed to the aſtringent acid in the gum arabic; a fact of which he is ſufficiently convinced by experience. Prof. GÖTTLING wiſhes that M. JUCH would give us a more ſatisfactory account of his experiments on this head, becauſe he could not obſerve the leaſt trace of an aſtringent acid in a gum perfectly tranſparent; or, at leaſt, could not diſcover the preſence of this acid, by treating gum arabic with a ſolution of the ſulphat of iron. "Is it not poſſible," aſks Prof. GÖTTLING, "that the nitrous acid here manifeſts a peculiar action on the gum; or that the gum is perhaps capable of depriving the mercurial calx of the nitrat of mercury, of a portion of its oxygen; and that it is thus reduced ſomewhat nearer to the metallic ſtate?" He farther adds, that, in the courſe of his experiments, when mixing the nitrat of mercury with ſugar, he once obſerved a ſimilar decompoſition take place, and that this circumſtance tends to corroborate his conjecture.—*Ibid.* p. 28.

---

Several methods of ſeparating the mineral alkali from common ſalt, and Glauber's ſalt, have lately been propoſed; two of which have been noticed in the "Journal for Manufactures, Commerce, and Faſhion," printed in German, for September, 1796. The firſt of theſe methods is that of Mr. HODSON, of Cheſter, who adviſes to treat common ſalt with powdered charcoal in a furnace of a peculiar conſtruction. According to his opinion, the phlogiſton of the carbon combines with the muriatic acid, which is thus reduced to a ſtate in which it may, without difficulty, be volatilized. The other method is that ſuggeſted by M. BONEUIL, of Liverpool, who treats Glauber's ſalt with charcoal powder and iron, in a proper temperature. From the carbon and the vitriolic acid of the Glauber's ſalt, ſulphur is ſaid to be produced, and this again is to combine with the iron, and form an artificial, ſilicious ſulphat. The diſengaged mineral alkali is then obtained by elixation, after it has been allowed to cryſtallize.

Prof. GÖTTLING remarks, that he has endeavoured, by experiments conducted on a ſmall ſcale, to aſcertain the practicability of theſe methods thods of difengaging the mineral alkali; but he found that the former was unfuccefsful, and the latter unprofitable. *Ibid.* p. 31.

---

A more effectual and ingenious method of *feparating the foda from fea-falt* has been propofed by Cit. Van Mons.—By mixing twenty parts of common falt, and thirteen parts of the carbonat of potafs, we obtain *digeftive falt*, and carbonat of foda. But as thefe falts can only be feparated with difficulty, Van Mons firft deprives the foda of its carbonic acid, by means of cauftic lime: the foda thus rendered cauftic remains uncryftallized in the ley, while the digeftive falt forms cryftals. The foda is then evaporated to drynefs, and afterwards, with the addition of powdered charcoal, expofed to a red heat, in a crucible. By this procefs, it again becomes mild, and fufceptible of cryftallization, fo that it may be completely purified. *Annals of Chemiftry* (in German) Vol. I. p. 40.

---

In a fimilar manner, *potafs may be effectually purified,* cryftallized, and deprived of all its foreign, faline ingredients, without lofing any part of its own fubftance. *Ibid.*

---

In the prefent dearth of medical information from the French Journals, whether tending to benefit or improve the practice of the healing art, we have not been able to fupply our readers with interefting extracts, efpecially as our fources of domeftic correfpondence have lately been multiplied beyond example. Among the numerous Effays on fubjects connected with medicine, we have, however, felected one, by J. J. Virey, of the Val de Grace, read to the Medical Society of Paris, on the 7th of Meffidor, in the 6th year, and entitled, "*General Re-*"*flections on the Aliment produced by the different Claffes of the Animal* "*Kingdom, and its Influence on the Human Body.*" Of this elaborate and curious paper we propofe to give our Readers a fatisfactory account, in a future Number of this Journal.

---

### *Domestic Intelligence.*

HAVING heard it repeatedly afferted, that Dr. Croft, of this City, had loft a child by the Vaccine Inoculation, we applied to him to learn the truth. He fhewed us a letter that he had written to Mr. Trye, of Gloucefter, in anfwer to the fame queftion, of which the following is an extract:

"Dear Sir,

"Hearing it is reported at Gloucefter, that I have loft a child of my own, under inoculation for the Cow-pox, I am convinced it will afford you pleafure to know that the only one of my children which was inoculated for the cow-pox, went through the difeafe in the moft defirable manner, and has ever fince been in perfect health. About thirty children inoculated from his arm had the difeafe equally well, and without any fubfequent illnefs, and I do believe that the whole number of children, dren, who were moſt of them inoculated by Mr. Knight, ſurgeon to the Duke of York, had not ſo much as one hour of indiſpoſition, or a ſingle puſtule, except on the arm. I trouble you with this for the purpoſe of preventing any miſrepreſentation that might tend to leſſen the value of Dr. Jenner's obſervations upon this important ſubject.

"I am, &c.

*Old Burlington Street,* "RICHARD CROFT."

*Oct.* 20, 1799.

---

A correſpondent recommends, and we cannot but agree in his ſentiments, that in order *to prevent, in ſome meaſure,* a continuance of the deluſion practiſed upon the credulous, by the venders of *quack-medicines,* an act of parliament ſhould be ſolicited, that no certificate of a cure by ſuch medicines be publiſhed in a newſpaper, or pamphlet, unleſs certified by an affidavit before a magiſtrate; and enjoining alſo, that, if any vender of Patent, or other Quack Medicines, ſhall publiſh a falſe affidavit, or any certificate of a cure, not atteſted upon oath, he ſhall be liable to a ſevere penalty; and that every affidavit be regiſtered in a particular office, previous to its publication. And if any ſuſpicious perſon ſhall appear before a magiſtrate, he ſhall be authorized to require the affirmation, or even the affidavit, of a reſponſible perſon, reſpecting the character of him who propoſes to ſwear to the cure.

---

The following Preamble to a Statute of Henry VIII. in favour of regular Phyſicians and Surgeons, is not inapplicable to the preſent *Age of Quackery*:—"Foraſmuch as the ſcience and cunning of Phyſic and "Surgery is daily, within this realm, exerciſed by a great multitude of "ignorant perſons, of whom the greater part have no inſight in the "ſame, nor in any other kind of learning; ſome alſo ken no letters on "the book; ſo far forth, that common artificers, as ſmiths, weavers, "and women boldly and accuſtomably take upon them great cures, in "which they partly uſe ſorcery and withcraft, partly apply ſuch me"dicines to the diſeaſe as be very noious, and nothing meet, to the high "diſpleaſure of God, great infamy of the Faculty, and the grievous "damage and deſtruction of divers of the King's people, &c. &c."

---

A Work on Inſanity is now preparing for the preſs, by John Reid, M. D.

---

A. and C. R. Aikin will reſume their evening courſe of Lectures on Chemiſtry, with its application to arts and manufactures, in the middle of November next. Their morning courſe will commence later in the year. Further particulars may be known by applying to Mr. C. R. Aikin, Surgeon, No. 4, Broad Street Buildings.

CRITICAL

